# Pancreatic alpha-cells from female mice undergo morphofunctional changes during compensatory adaptations of the endocrine pancreas to diet-induced obesity

**DOI:** 10.1038/srep11622

**Published:** 2015-06-25

**Authors:** Beatriz Merino, Paloma Alonso-Magdalena, Mónica Lluesma, Patricia Ñeco, Alejandro Gonzalez, Laura Marroquí, Marta García-Arévalo, Angel Nadal, Ivan Quesada

**Affiliations:** 1 Instituto de Bioingeniería, Universidad Miguel Hernández, Elche, Spain; 2 CIBER de Diabetes y Enfermedades Metabólicas Asociadas (CIBERDEM), Spain

## Abstract

Obesity is frequently associated with insulin resistance. To compensate for this situation and maintain normoglycaemia, pancreatic beta-cells undergo several morphofunctional adaptations, which result in insulin hypersecretion and hyperinsulinaemia. However, no information exists about pancreatic alpha-cells during this compensatory stage of obesity. Here, we studied alpha-cells in mice fed a high-fat diet (HFD) for 12 weeks. These animals exhibited hyperinsulinaemia and normoglycaemia compared with control animals in addition to hypoglucagonaemia. While the *in vivo* response of glucagon to hypoglycaemia was preserved in the obese mice, the suppression of glucagon secretion during hyperglycaemia was impaired. Additionally, *in vitro* glucagon release at low glucose levels and glucagon content in isolated islets were decreased, while alpha-cell exocytosis remained unchanged. Assessment of morphological parameters revealed that alpha-cell area was reduced in the pancreas of the obese mice in association with alpha-cell hypotrophy, increased apoptosis and decreased proliferation. HFD feeding for 24 weeks led to significant deterioration in beta-cell function and glucose homeostasis. Under these conditions, the majority of alpha-cell changes were reversed and became comparable to controls. These findings indicate that pancreatic compensatory adaptations during obesity may also involve pancreatic alpha-cells. Additionally, defects in alpha-cell function during obesity may be implicated in progression to diabetes.

Glucagon secretion plays a key role in glucose homeostasis. This hormone activates gluconeogenesis and glycogenolysis, which enhances hepatic glucose production, allowing for the restoration of plasma glucose levels from a hypoglycaemic state. In contrast, pancreatic alpha-cell secretion is inhibited by elevated plasma glucose levels. Thus, insulin from beta-cells and glucagon from alpha-cells, which respond reciprocally to plasma glucose changes, constitute a bihormonal system for the adequate control of glycaemia^1^. It has been documented that impaired alpha-cell function may occur in diabetes. For instance, the response of alpha-cells to low glucose levels may be disrupted in this disease, restricting one of the first defences against hypoglycaemia^2^. Additional alterations include hyperglucagonaemia and a lack of glucagon suppression at high glucose levels, which may contribute to hyperglycaemia in these patients. In this regard, the inhibition of either glucagon release or its action has been used as an approach to decrease hyperglycaemia in experimental and clinical diabetes^1^. Recently, it has been reported that pancreatic alpha-cells can dedifferentiate to beta-cells under stress conditions, which may be of high significance in cell therapy^3^,^4^. These therapeutic implications have renewed interest in the biology of alpha-cells and their contribution to diabetes.

Obesity and overweight, which are frequently associated with insulin resistance, are important risk factors for the development of type 2 diabetes^5^. Insulin resistance increases the insulin demand of the organism. It is well accepted that in response to these conditions, beta-cells undergo several morphofunctional compensatory adaptations, which lead to enhanced insulin secretion and hyperinsulinaemia to maintain normoglycaemia^6^,^7^. However, when beta-cell adaptations fail to compensate for these conditions, impaired glucose homeostasis can occur, leading to hyperglycaemia and type 2 diabetes. In later stages, gradual losses of beta-cell mass and function may further deteriorate glucose homeostasis^8^. Thus, the compensation for insulin resistance in these cells in obesity is crucial to avoid eventual progression to hyperglycaemia and type 2 diabetes.

In contrast with beta-cells, knowledge about the behaviour of pancreatic alpha-cells in obesity is scarce. Although few reports have described alterations in both alpha-cell function and plasma glucagon levels in obese individuals and animals, most studies have been performed at stages during which glucose homeostasis and beta-cell function may be already deteriorated^9^,^10^,^11^,^12^. However, there is no information about alpha-cells during the stages of islet compensation for obesity, in which normoglycaemia is maintained. Therefore, in the present study, we examined the behaviour and morphofunctional features of pancreatic alpha-cells as well as glucagon release during the compensatory adaptation of the islet in a model of high-fat diet-induced obesity.

## Methods

### Animals, diets, and plasma parameters

All experimental protocols were approved by the Animal Ethics Committee of Miguel Hernández University according to national regulations (Reference number: UMH.IB.IQM.01.13). All the methods were carried out in accordance with the approved guidelines. Experiments were performed using C57BL/6J mice. After weaning, 21-day-old female pups were fed for 12 or 24 weeks with either of the following diets obtained from Research Diets (New Brunswick, NJ): a normal diet (ND; 10% fat, 20% protein, and 70% carbohydrates; reference D12450B) or a high-fat diet (HFD; 60% fat, 20% protein, and 20% carbohydrates; reference D12492). The animals were housed in groups of 3 at 22 °C and a light cycle of 12 h (8:00 am to 8:00 pm).

### Islet isolation and cell culture

Mice were killed at the end of the dietary treatments by cervical dislocation, and islets were then isolated by collagenase digestion as previously described^13^. In some experiments, isolated islets were dispersed into single cells by trypsin enzymatic digestion and then cultured overnight at 37 °C in RPMI 1640 (Sigma, Madrid, Spain) supplemented with 10% foetal calf serum, 100 IU/mL penicillin, 0.1 mg/mL streptomycin, and 11 mM D-glucose^13^. Except when indicated, all experiments were conducted at 37 °C.

### Plasma measurements

Blood glucose was measured from tail vein blood using an automatic glucometer (Accu-Chek Compact plus model GT, Roche, Mannhein, Germany). Blood samples for glucagon measurement were collected in aprotinin (20 mg/L) to protect them from proteolysis. Plasma glucagon and insulin concentrations were determined by ELISA (Gentaur, Kampenhout, Belgium and Crystal Chem Inc., IL 60515, USA, respectively). Plasma somatostatin and glucagon-like peptide-1 (GLP-1) levels were determined by ELISA (Phoenix Pharmaceuticals Inc., Karlsruhe, Germany and Alpco, Barcelona, Spain, respectively). Blood samples for somatostatin and GLP-1 determination were collected in the presence of aprotinin (20 mg/L) or aprotinin and dipeptidyl peptidase-4 inhibitor (DPP4-010 Merck, Madrid, Spain), respectively. The HOMA-IR was also calculated as an indicator of insulin resistance: [fasted plasma glucose (mg/dL) * fasted plasma insulin (mU/L)]/405^14^.

### Glucagon secretion and content measurements

Freshly isolated islets were left to recover in the isolation medium for 2 h in an incubator^15^. After recovery, groups of 20 islets were transferred to 300 μL of a buffer solution containing (in mM): 140 NaCl, 4.5 KCl, 2.5 CaCl_2_, 1 MgCl_2_, 20 HEPES and 0.5 glucose, with final pH of 7.4, supplemented with 0.1% of BSA. Then, the islets were incubated for 1 h at 37 °C. Next, the groups were transferred to another buffer solution with the same composition and corresponding glucose concentrations and incubated for 1 h. The islets were then incubated at room temperature for 3 min and cooled for 15 min on ice. The medium was collected, and the glucagon concentration was measured in duplicate samples by ELISA using a Glucagon EIA Kit #YK090 Gentaur (Kampenhout, Belgium). To determine the glucagon content, the islets that were grouped into batches of 20 were handpicked and incubated overnight in an ethanol/HCl buffer at 4 °C. At the end of the incubation period, the buffer was removed, and the glucagon content was analysed. The protein concentration was measured by the Bradford dye method.

### Immunocytochemistry, immunohistochemistry and alpha-cell mass

The pancreas samples were removed and fixed overnight in 4% paraformaldehyde. Subsequently, the pancreatic tissue was embedded in paraffin, and sections were prepared and stained for alpha-cell identification, according to previous reports^16^,^17^. For the quantification of alpha-cell area, sections were viewed at 20x magnification. The islet cross-sectional area, total pancreatic area and alpha-cell sizes were measured using Metamorph Analysis Software (Nashville, TN, USA). Two to three pancreas sections separated by 200 μm were measured per animal. All sections were obtained transversely and identically processed for all samples. The whole area of each section was measured as well as all the islets included in each section. To perform immunocytochemistry, glucagon-containing cells were identified with polyclonal anti-glucagon rabbit antibodies (1:100; Monosan) as previously described^18^. To measure proliferation, mice were administered intra-peritoneal injections of BrdU (100 μg/g) every 12 h for 3 consecutive days before sacrifice, as previously described^18^. Pancreatic tissue was collected, fixed, and processed as described above. After dehydration, the sections were heated to 100 °C in the presence of citrate buffer (10 mM) for 20 min and immersed in 2 N HCl for 5 min, followed by incubation in a 0.1 M borax solution for 10 min at RT and a wash step with phosphate-buffered saline (PBS). The slides were then blocked by incubation for 1 h in 3% bovine serum albumin in PBS. Next, the samples were incubated with antibodies for glucagon (1:100; Monosan) and BrdU (1:100, monoclonal; DAKO, Barcelona, Spain) overnight at 4 °C. Proliferation was also analyzed by immunohistochemistry of proliferating cell nuclear antigen^17^ (PCNA; 1:2400; Cell Signaling, Technology, Danvers, MA). After incubation with the appropriate secondary antibodies (Alexa Fluor, Invitrogen, Barcelona, Spain), the sections were stained with Hoechst 33342 (Invitrogen, Barcelona, Spain). Apoptosis was quantified in the pancreas sections by the TUNEL technique^17^,^18^. After incubation with secondary antibodies, the sections were mounted using ProLong Gold Antifade Reagent (Invitrogen, Barcelona, Spain). Images were acquired from double-stained sections. BrdU or TUNEL-positive nuclei were scored only in cells that were also positive for glucagon. Alpha-cell sizes were also measured by analysis of the cross-sectional areas of glucagon-positive islet cells dispersed in primary culture.

### Patch-clamp measurement

Exocytosis was monitored in cells within intact fresh islets by standard whole-cell configuration and by recording cell capacitance changes through the sine + DC mode of the Lock-In amplifier included in Patch Master software. For these experiments, a pipette solution was prepared, containing the following components (in mM): 125 glutamic acid, 10 NaCl, 10 KCl, 1 MgCl_2_, 0.05 EGTA, 3 Mg-ATP, 0.1 cAMP, and 5 HEPES (pH 7.1 with KOH). A bath solution was also prepared with the following components (in mM): 118 NaCl, 5.6 KCl, 20 tetraethylammonium-Cl, 1.2 MgCl_2_, 2.6 CaCl_2_, 5 HEPES, and 5 D-glucose (pH 7.4 with NaOH)^19^. Only experiments showing stable and low access resistance and small leak currents were evaluated. The seal resistance value was typically >3 MΩ. No differences were allowed between the ND and HFD groups in any of these parameters. The experiments were carried out at physiological temperature (34 °C–36 °C). Alpha-cell identity was established by a double method. First, the characteristic voltage-dependent inactivation of the Na^+^-current was evaluated by a double-step protocol, in which the cell was held at -70 mV, and conditioning pulses between −150 and 0 mV were applied before a test pulse to +10 mV^20^. Then, the TEA-resistant transient K^+^ current sensitive to 4-AP (A-current) was evaluated by voltage-clamp depolarizations from −70 to −10 mV^20^. Additionally, some cells were tracked by immunocytochemistry through injection of biocytin (0.5 mg/mL) via a recording electrode^20^. Cells that were positive for A-currents were also positive for anti-glucagon staining.

### Statistical analysis

The data are presented as the mean ± SE. Student’s *t* test or one-way ANOVA with Bonferroni correction was performed with a level of significance of *p* < 0.05.

## Results

### Mice fed HFD for 12 weeks exhibit low levels of plasma glucagon

After 12 weeks of high-fat diet (HFD) consumption, the obese mice exhibited increased body weight compared with the animals fed a normal diet (ND) (Table 1). As shown in a previous study using this model^21^, the HFD mice displayed fed and fasting hyperinsulinaemia and a higher HOMA index compared with the controls (Table 1). Fed and fasting glycaemia were comparable in both groups and were within the physiological range in this study (Table 1). When subjected to an intraperitoneal glucose tolerance test (ipGTT), the obese mice presented slightly elevated glucose levels at 15 and 30 min; however, levels were similar at the beginning and after 2 h of the glucose load (Supplementary Fig. 1). We have previously shown in this experimental model that HFD animals exhibit decreased peripheral insulin sensitivity after an intraperitoneal insulin tolerance test (ipITT) as well as increased plasma insulin responses to a glucose load^21^. Overall, the current and previous results for this model of HFD-induced obesity indicate that these animals develop mild insulin resistance and that plasma glucose levels remain within a physiological range at the expense of increased plasma insulin levels. As previously shown in the same HFD-induced obesity model, the hyperinsulinaemia in this study resulted from structural and functional beta-cell compensatory adaptations^21^. Thus, glucose homeostasis was preserved in the HFD mice.

**Figure 1:**
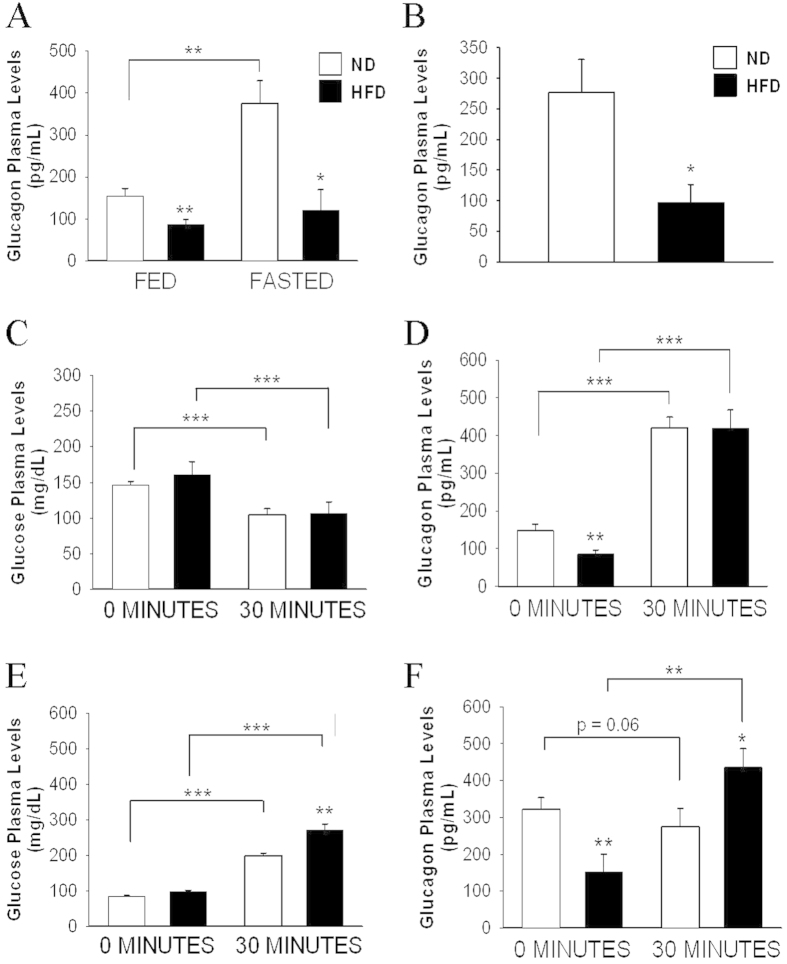
Plasma glucagon levels and *in vivo* glucagon responses in mice fed HFD for 12 weeks and in controls. **A**: Glucagonaemia in ND and HFD mice in the fed state and after 6 h of fasting (n = 12 mice, fed group; and n = 5 mice, fasted group). **B**: Glucagonaemia after 12 h of fasting (n = 6 mice each group). **C**,**D**: Glycaemia and glucagonaemia measured in the fed state at 0 and 30 min after one dose of 0.75 U/kg insulin (n = 6 mice each group). **E**,**F**: Glycaemia and glucagonaemia measured in the fasting state (12 h) at 0 and 30 min after one dose of 2 g/kg glucose (n = 6 mice each group). ND: normal diet, white bars; and HFD: high-fat diet, black bars. Statistically significant: *p < 0.05; **p < 0.01; and ***p < 0.001.

Examination of plasma glucagon levels during this islet compensatory stage in the obese animals revealed the presence of hypoglucagonaemia in the fed state and after fasting for 6 and 12 h (Fig. 1A,B). The *in vivo* alpha-cell response to hypoglycaemia was analysed following intraperitoneal insulin injection (Fig. 1C,D). Thirty minutes after insulin injection, plasma glucose levels decreased in the ND and HFD mice to the same extent (Fig. 1C). Plasma glucagon levels also displayed similar increases in both groups (Fig. 1D). When challenged with an intraperitoneal glucose load, while the controls exhibited a slight decrease in glucagon release, plasma glucagon levels were increased in the obese animals (Fig. 1E,F). Thus, these experiments indicate that *in vivo* plasma glucagon levels are decreased in HFD mice under basal conditions and are impaired in response to hyperglycaemia. While no differences were found in the plasma somatostatin concentrations between both groups (data not shown), the plasma GLP-1 levels in the fed state were higher in the HFD mice compared with the control mice (1.45 ± 0.31 pM for ND; 3.46 ± 0.68 pM for HFD; p < 0.05).

### Glucagon secretion and content are altered in mice fed HFD for 12 weeks

**Figure 2:**
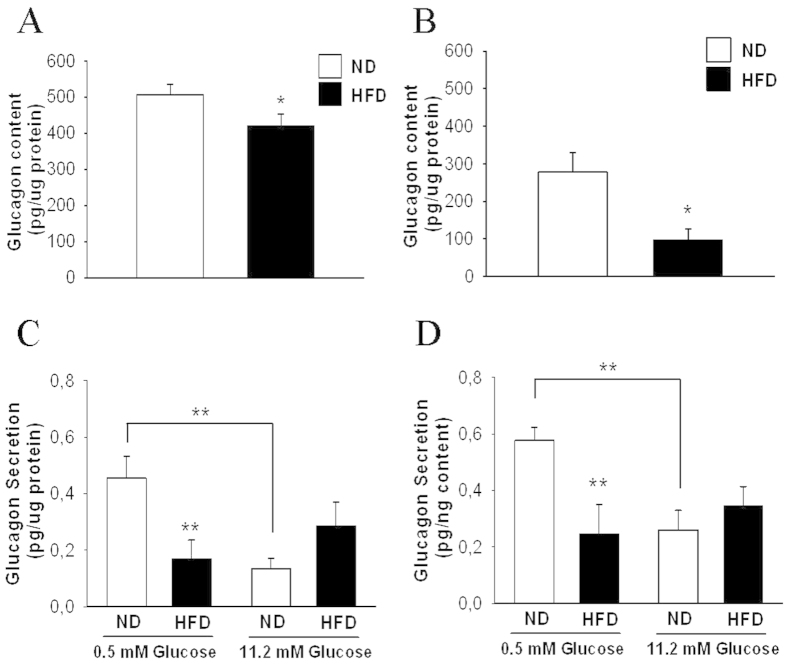
Glucagon content and secretion in isolated pancreatic islets of mice fed HFD for 12 weeks and controls. **A**,**B**: Glucagon content normalized to the total protein level in isolated islets obtained from mice in the fed state (A) (ND, n = 6 mice; HFD, n = 9 mice; 140 islets per treatment) or fasting state (B) (ND, n = 10 mice; HFD, n = 9 mice; 140 islet per treatment). **C**, **D**: Glucagon release from isolated islets after static incubation with different glucose concentrations was normalized to the total protein level (C) (ND, n = 12 mice; and HFD, n = 12 mice) or glucagon content (D) (ND, n = 12 mice; and HFD, n = 12 mice). ND: normal diet, white bars; and HFD: high-fat diet, black bars. Statistically significant: *p < 0.05; and **p < 0.01.

**Figure 3:**
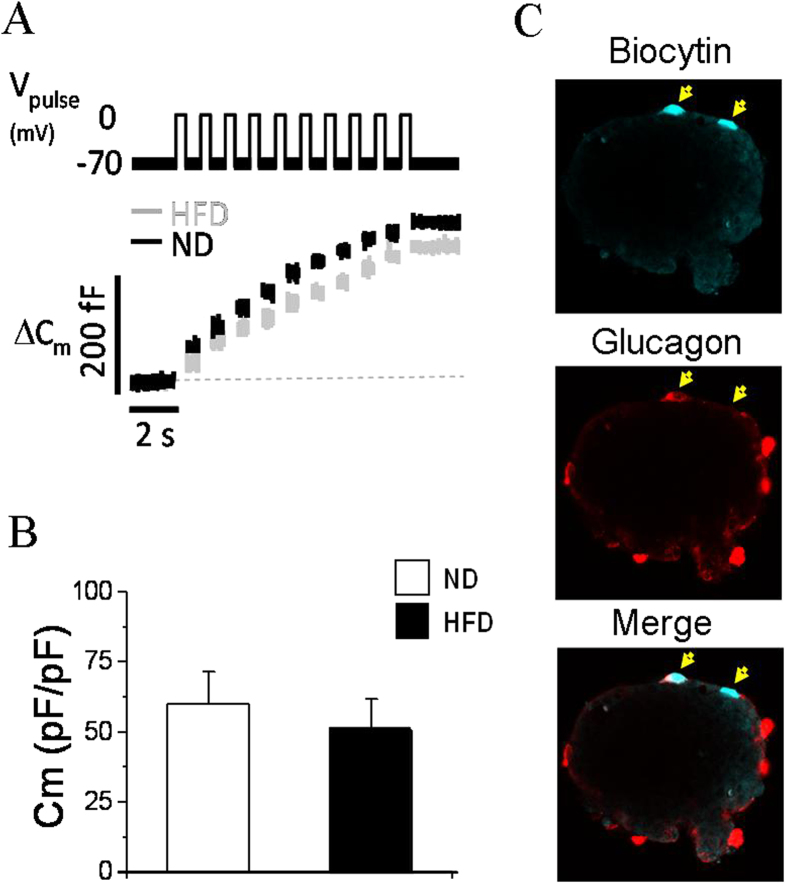
Exocytosis in pancreatic alpha-cells within intact islets in obese and lean mice. **A**: Representative capacitance measurements elicited by ten consecutive depolarization pulses of 500 ms from −70 to 0 mV in alpha-cells within intact islets. **B**. Average capacitance increases normalized to cell size (ND, n = 7 cells; and HFD, n = 6 cells). No significant differences were found. **C**: Representative glucagon-positive immunoidentification of one cell from two patched cells injected with biocytin.

To determine whether the above-mentioned alterations were due to an intrinsic failure in islet secretion, we assessed glucagon release in isolated pancreatic islets. Glucagon protein content was reduced in the HFD animals under both the fed and fasting conditions (Fig. 2A,B). Exposure of pancreatic islets to glucose concentrations known to promote *in vitro* glucagon release near the maximal (0.5 mM) and minimal (11 mM) levels^22^ resulted in impaired glucagon secretion at 0.5 mM glucose in the obese mice (Fig. 2C,D). Analysis of the capacitance responses to depolarization pulses in identified alpha-cells within the pancreatic islets revealed no differences in exocytosis between the two groups (Fig. 3). Thus, the impaired glucagon secretion at 0.5 mM glucose in the pancreatic islets of the HFD mice may have been due to alterations other than those affecting the exocytotic process.

### Mice fed HFD for 12 weeks display a decrease in alpha-cell mass, size and proliferation and increased apoptosis

**Figure 4:**
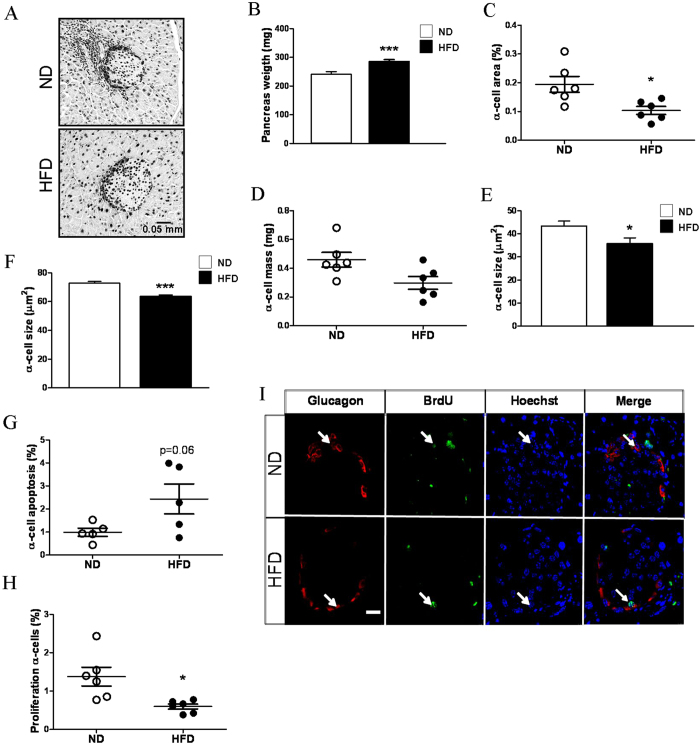
Alpha-cell mass in mice fed HFD for 12 weeks and in controls. **A**: Images of islets from pancreas sections stained for glucagon and haematoxylin. **B**: Pancreas weight (n = 6 mice each group). **C**: Alpha-cell area (n = 6 mice each group). **D**: Alpha-cell mass (n = 6 mice each group). Analysis of alpha-cells was performed using 299 islets from the ND mice and 324 islets from the HFD mice. **E**. Sizes of glucagon-positive cells in the pancreas sections (ND, n = 2196 cells from 35 slides; and HFD, n = 3601 cells from 34 slides; n = 5 mice each group). **F**. Sizes of glucagon-positive islet cells isolated in primary culture (ND, n = 201 cells; and HFD, n = 191 cells, from 7 and 8 mice, respectively). **G**: Percentage of apoptosis in glucagon-positive cells (ND, n = 2421 cells; HFD, n = 3851 cells; n = 5 mice each group). **H**: Proliferation measured as a percentage of glucagon-positive cells that incorporated BrdU (ND, n = 2972 cells; and HFD, n = 4854 cells; n = 6 mice each group). **I**: Representative images of islets stained for glucagon (red), BrdU (green) and Hoechst (blue). ND: normal diet, white bars; and HFD: high-fat diet, black bars. Statistically significant: *p < 0.05; and ***p < 0.001.

We have previously demonstrated in the same HFD-induced obesity model that mice subjected to a HFD for 12 weeks present augmented beta-cell mass and size^21^. Here, we showed that under these conditions, the alpha-cell area was decreased by ~50% in the HFD animals (Figs 4A–C). The absolute alpha-cell mass in grams considering the pancreas weight showed a trend of decrease by ~35% in the HFD mice (Fig. 4D). These morphological alterations were supported by various changes in alpha-cell size, apoptosis and proliferation. Alpha-cell size was found to be smaller in the HFD animals in the pancreas sections and under *in vitro* conditions (Fig. 4E,F). Apoptosis was increased in alpha-cells from the HFD mice (Fig. 4G). Proliferation was assessed in the pancreases as the proportion of BrdU-positive alpha-cells, revealing a decrease in this parameter in the obese mice (Fig. 4H,I). Similar results, including reductions in alpha-cell area and proliferation and an increase in apoptosis were observed in a different set of mice (Supplementary Fig. 2). Moreover, experiments with a different proliferation marker (PCNA) also indicated a decrease in alpha-cell proliferation in the obese mice (Supplementary Fig. 3).

### Effects of HFD consumption for 24 weeks on glucagon release and pancreatic alpha-cells

It has been shown that prolonged exposure to a HFD can result in the insufficient compensatory adaptation of beta-cells to obesity and that this situation can progress to impaired beta-cell function and type 2 diabetes^23^. To analyse whether prolonged exposure to a HFD can affect the alpha-cell changes observed at 12 weeks similar to what was found for the beta cells, the animals were fed a HFD for 24 weeks. After this period, the fed and fasting insulin levels were markedly elevated in the obese animals (Table 2). Despite this hyperinsulinaemia, fed and fasting hyperglycaemia were present, and glucose tolerance was deteriorated (Table 2; Supplementary Fig. 1). All of these findings indicate that the adaptations of beta-cells that occurred for the maintenance of normoglycaemia after 12 weeks of HFD consumption were not present after 6 months of the intake of this diet. Consistent with this deterioration in beta-cell function, hyperglycaemia became evident.

**Figure 5:**
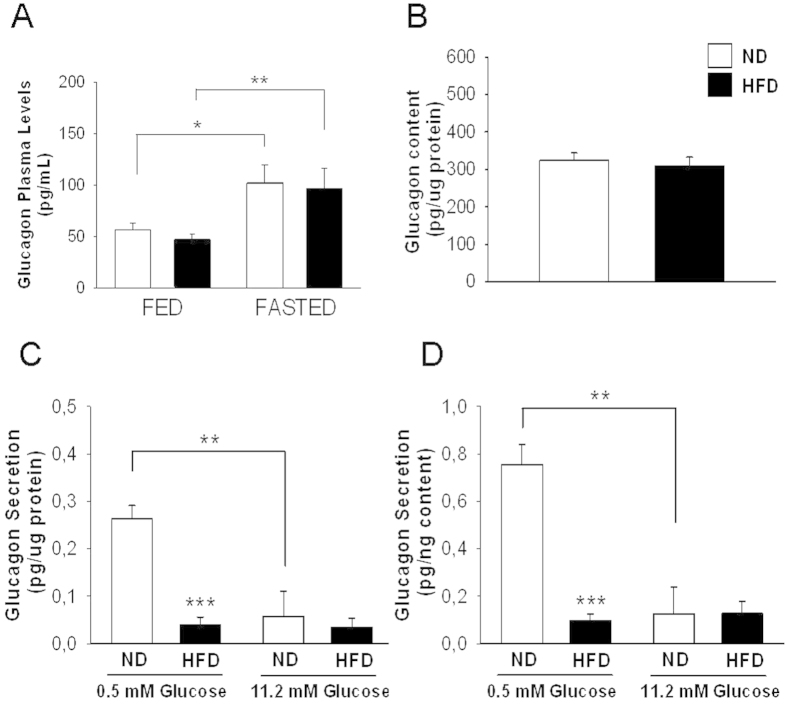
Glucagon plasma levels and secretion after 24 weeks of HFD consumption. **A**: Glucagon plasma levels measured in mice in fed and 12 h-fasted states (n = 10–20 mice for each group). **B**: Glucagon content concentrations normalized to the total protein level (ND, n = 10 mice; HFD, n = 12 mice; 140 islets per treatment). **C**,**D**: Glucagon secretion from isolated islets after static incubations with different glucose concentrations normalized to the total protein (C) or to total glucagon content (D) (ND, n = 10 mice; and HFD, n = 9 mice). ND: normal diet, white bars; and HFD: high-fat diet, black bars. Statistically significant: **p < 0.01; and ***p < 0.001.

**Figure 6:**
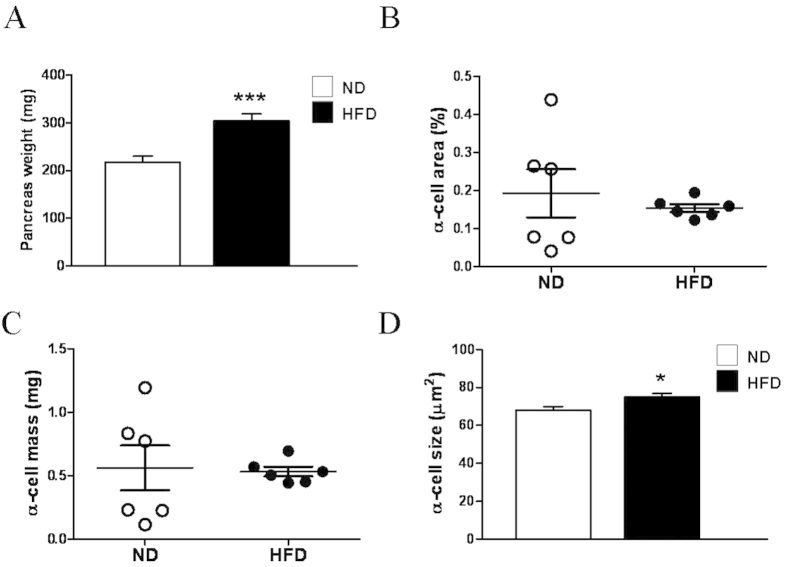
Alpha-cell masses after HFD consumption for 24 weeks. **A**: Pancreas weight (n = 6 mice each group). **B**: Alpha-cell area (n = 6 mice each group). **C**: Alpha-cell mass (n = 6 mice each group). Analysis of alpha-cells was performed using 318 islets from the ND mice and 255 islets from the HFD mice. **D**: Sizes of glucagon-positive cells isolated from culture (ND, n = 50 cells; and HFD, n = 112 cells, from 10 and 9 mice, respectively). ND: normal diet, white bars; and HFD: high-fat diet, black bars. Statistically significant: *p < 0.05; and **p < 0.01.

After 24 weeks, most alpha-cell parameters in the HFD mice returned to the control values. For example, the plasma glucagon levels (Fig. 5A) as well as the glucagon content in the pancreatic islets (Fig. 5B) were similar in both groups. In contrast, the impaired glucagon secretion at 0.5 mM glucose persisted in the pancreatic islets after 24 weeks of HFD consumption (Fig. 5C,D). Analysis of alpha-cell mass after 6 months revealed an absence of differences between the obese and control mice (Fig. 6). Instead of the hypotrophy shown in Fig. 4, alpha-cell size was increased (Fig. 6D). Thus, it seems that most of the alpha-cell changes that developed during the compensation of beta-cells to 12 weeks of HFD consumption were transitory. After prolonged exposure to the HFD, plasma glucagon levels and the majority of alpha-cell morphological parameters returned to the normal values, coinciding with beta-cell deterioration and impaired glucose homeostasis.

## Discussion

The compensatory response of beta-cells in obesity has been well studied. The augmented insulin demand imposed by insulin resistance leads to an increase in beta-cell mass and function to elevate plasma insulin levels and maintain normoglycaemia^6^,^7^. However, information about the potential adaptations of pancreatic alpha-cells at this stage of obesity is absent. Here, we used a previously characterized HFD-induced obesity model^21^ to study alpha-cell morphofunctional characteristics during this adaptive phase. In accordance with previous studies^21^, our animals, which were fed a HFD for 12 weeks, were hyperinsulinaemic and exhibited normal fed and fasting glycaemia (Table 1). The initial and final glucose levels were similar in the HFD and control mice after ipGTT. All of these features indicate that our HFD animals are compatible with a model of obesity with normal glycaemia and increased plasma insulin levels. We and others have shown that in this compensated hyperinsulinaemic stage, augmented beta-cell mass and function are present^5^,^6^,^7^,^21^. This HFD-induced obesity model was developed in female mice as previously reported^21^. It has been indicated that females are protected against HFD-induced obesity, insulin resistance and progression to type 2 diabetes^24^,^25^. Deterioration of glucose tolerance during HFD-induced obesity may occur more slowly in female mice, given that similar dietary protocols result in deteriorated beta-cell function and hyperglycaemia in male mice^23^. Thus, we analysed alpha-cells and glucagon responses in HFD-treated female mice, which represent a good model for the study of obesity in the prediabetic state characterized by normoglycaemia, as previously shown^21^,^26^.

While several alterations have been described in alpha-cells in type 2 diabetes^2^, the functions of these cells in prediabetic states, such as that explored in this study, are less clear. Remarkably, in this islet compensatory stage, hypoglucagonaemia was observed in the fasted and fed HFD animals (Fig. 1). Although few studies have measured plasma glucagon levels in HFD-induced obese mice, this parameter has been reported to be normal^9^,^27^,^28^,^29^, elevated^9^ and decreased^30^. From these studies, only one was performed in female mice^29^. Some of the discrepancies among these studies may be associated with the different dietary treatments with regard to time and composition as well as the obesity stages analysed. A similar situation has been reported in humans. In obese subjects, plasma glucagon levels and alpha-cell function have also been shown to be normal, augmented and decreased^10^,^11^,^12^,^31^,^32^,^33^,^34^. Except one study performed in women, the rest of reports were conducted in mixed populations of men and women^31^.These differences may also be caused by variations in the glycaemic profiles of the subjects analysed. Indeed, hyperglucagonaemia in obesity has been associated with impaired glucose tolerance and type 2 diabetes in cases in which glucose homeostasis has already deteriorated^10^,^11^,^31^,^33^. In contrast, the response of alpha-cells to alanine has been found to be decreased in obese subjects with normal glucose tolerance^12^. Specific studies of alpha-cells during the different events of islet compensation are required in both humans and animals to further understand this process.

While the *in vivo* response to hypoglycaemia was well preserved in the HFD mice, the suppression of glucagon release at high glucose levels was disrupted (Fig. 1). In agreement with our results, the impaired suppression of glucagon secretion during hyperglycaemia has been described in both HFD-treated mice^27^ and obese subjects^10^,^35^. In type 2 diabetes, alterations in alpha-cells during both hyper- and hypoglycaemia can also be present^2^. Thus, our findings suggest that this alpha-cell defect may appear in obesity with normal glucose tolerance in the eventual progression to type 2 diabetes. The lack of suppression of glucagon release has been attributed to either alpha-cell insulin resistance or impaired glucose sensing by the nervous system or by the alpha-cells themselves^1^,^36^; however, the nature of this *in vivo* defect in obesity remains to be determined.

As discussed in the previous paragraph, the *in vivo* responses of alpha-cells to plasma glucose changes are regulated by several factors, with a major role of autonomic inputs^37^. Notwithstanding, the examination of *in vitro* glucagon release indicated an islet defect in glucagon secretion at 0.5 mM glucose (Fig. 2). Although non-physiological, this glucose concentration leads to a maximal secretory response in isolated islets, allowing for the better detection of potential defects in secretion^22^. Additionally, at this glucose concentration, paracrine effects due to beta or delta-cell stimulation should be absent^1^,^22^. We found that this secretory alteration, which occurred in animals fed HFD for 12 weeks, persisted after 24 weeks of HFD consumption (Fig. 5). When depolarization-evoked exocytosis was compared in alpha-cells within the islets of both models, no differences were found (Fig. 3). In this experimental setting, only patched cells should be stimulated by depolarizing pulses; thus, paracrine effects are unlikely to occur, indicating that the disrupted glucagon secretion at 0.5 mM glucose may be associated with defects distal to alpha-cell exocytosis, such as steps involved in glucose sensing^38^ or in the stimulus-secretion coupling of these cells^1^,^39^. Notably, these very low glucose concentrations are not reached under *in vivo* conditions, even after insulin-induced hypoglycaemia (Fig. 1E). Additionally, as shown in Fig. 1, it seems that *in vivo* regulatory mechanisms^37^ may prevail or bypass the defects observed at 0.5 mM glucose. Indeed, in addition to the glucose-sensing capacity of pancreatic alpha-cells^40^, *in vivo* glucagon secretion in response to hypoglycaemia and plasma glucose changes are largely regulated by neural and adrenergic inputs^36^,^37^,^41^.

In addition to the functional changes in the alpha-cells, their morphological characteristics were also affected by 12 weeks of HFD consumption. The reduced alpha-cell mass observed in the obese mice was probably a consequence of diminished alpha-cell size and proliferation as well as increased apoptosis (Fig. 4). The low plasma glucagon levels under resting conditions for both the fasting and fed states (Fig. 1A) may have also resulted, at least partly, from these structural features. It has been previously documented that male mice fed a HFD for 18 weeks exhibit a trend of increased alpha-cell mass but normal circulating glucagon levels^27^. In male and female baboons, alpha-cell mass also tend to be augmented with the duration and severity of obesity^42^. Both obese and non-obese subjects with type 2 diabetes (mixed groups of men and women) may display increased alpha-cell mass in absolute values or relative to those of beta-cells^1^,^2^,^43^. However, our study is the first to specifically analyse the compensatory adaptations during obesity to maintain a normoglycaemic state, showing a decrease in the alpha-cell population. Given that insulin and glucagon seem to increase alpha-cell proliferation^44^, the diminished proliferative values observed here may be associated with the lower plasma glucagon levels (Fig. 1A) and are likely related to alpha-cell insulin resistance, as has been suggested to occur in type 2 diabetes^45^ and after long-term insulin exposure^46^.The lack of suppression of glucagon release (Fig. 1F) may also be indicative of alpha-cell insulin resistance. In agreement with other diet-induced obesity models^47^,^48^, we found increased plasma GLP-1 levels in the HFD mice. Because GLP-1 inhibits glucagon function^49^ and may decrease alpha-cell mass^50^, this hormone may be involved in the alterations observed in this study. In any case, numerous neural, metabolic and hormonal factors present during obesity could affect alpha-cells^1^,^36^. Some of these factors as well as the glycaemic environment are also subjected to changes as obesity evolves. Thus, a complex interplay of signals is probably affecting alpha-cells.

Interestingly, when the HFD was extended by up to 24 weeks, the plasma glucagon concentrations along with the majority of the morphological parameters were similar to the control values. In contrast with the previous compensatory stage, during which mice exposed to the HFD for 12 weeks exhibited hyperinsulinaemia and normoglycaemia, these later alpha-cell changes after HFD consumption for 24 weeks coincided with the deterioration of beta-cell function and glucose homeostasis (Supplementary Fig. 1). Thus, alpha-cells exhibit notable plasticity during HFD intake, as has been demonstrated for beta-cells. We suggest that in addition to enhancements in beta-cell function and mass during compensation to maintain normoglycaemia in obese animals (12 weeks of HFD feeding^21^), the down-regulation of alpha-cells and glucagon levels would also promote glucose homeostasis. In later stages of obesity, when glucose intolerance and beta-cell deterioration were present (after 24 weeks of HFD feeding), the previous alpha-cell changes could no longer be maintained. At more advanced stages during progression to type 2 diabetes, when beta-cell malfunction and hyperglycaemia are markedly increased, it has been reported that alpha-cell mass and function is up-regulated, contributing to hyperglycaemia^2^,^44^,^51^. While the majority of parameters were similar to the control values when the animals were fed the HFD for 24 weeks, the secretory defects observed in the isolated islets persisted in the obese mice. Thus, as mentioned above, it seems that *in vivo* regulation (i.e., autonomic and adrenal inputs) may bypass this islet alteration to maintain normal plasma glucagon levels in mice fed a HFD for 24 weeks. Isolated islets from type 2 diabetic donors exhibit altered glucagon release in response to glucose and also seem to secrete less glucagon than controls, particularly at low glucose levels^40^. Thus, high plasma glucagon levels in type 2 diabetes may result from an up-regulation of the *in vivo* control of islet glucagon secretion and/or increases in alpha-cell mass in the pancreas, as previously suggested^2^,^43^,^51^. In this regard, it would be interesting to further characterize alpha-cells during the progression from normoglycaemia and moderate hyperglycaemia (as in the animal models described here) to later stages of overt hyperglycaemia and type 2 diabetes because their function may change along with this progression, as has been shown for beta-cells^7^.

Overall, the present findings suggest that in addition to beta-cells, alpha-cells may also participate in adaptations to obesity. Additionally, the loss of these alpha-cell adaptations or changes (similar to beta-cells) may be implicated in progression to hyperglycaemia. The mechanisms regulating this alpha-cell plasticity are still unknown and merit further investigation. Whether these adaptations could be manipulated, it would be of interest for the design of therapeutic strategies for hyperglycaemia and diabetes.

## Additional Information

**How to cite this article**: Merino, B. *et al.* Pancreatic alpha-cells from female mice undergo morphofunctional changes during compensatory adaptations of the endocrine pancreas to diet-induced obesity. *Sci. Rep.* **5**, 11622; doi: 10.1038/srep11622 (2015).

## Supplementary Material

Supplementary Information

## Figures and Tables

**Table 1:** Body weights and plasma parameters in fed and fasting states in ND and HFD mice after feeding for 12 weeks (ND, n = 13 mice; and HFD, n = 11 mice).

12 weeks	ND	HFD
**Final weight (g)**	20.85 ± 0.13	28.01 ± 0.23***
**Fed glycaemia (mg/dL)**	165 ± 5.45	172 ± 3.74
**Fed insulin serum (ng/mL)**	0.83 ± 0.15	1.44 ± 0.15**
**Fasted glycaemia (mg/dL)**	104.83 ± 10.03	113.55 ± 4.97
**Fasted insulin serum (ng/mL)**	0.75 ± 0.08	1.24 ± 0.01**
**HOMA IR**	1.08 ± 0.21	1.85 ± 0.05*

Statistically significant: *p < 0.05; **p < 0.01; and ***p < 0.001. ND, normal diet; and HFD, high-fat diet.

**Table 2:** Body weights and plasma parameters in fed and fasting states in ND and HFD mice after feeding for 24 weeks (ND, n = 6 mice; and HFD, n = 9 mice).

24 weeks	ND	HFD
**Final weight (g)**	22.73 ± 0.59	43.67 ± 1.06***
**Fed glycaemia (mg/dL)**	155.8 ± 7.51	170.33 ± 2.93*
**Fed insulin serum (ng/mL)**	1.46 ± 0.23	2.64 ± 0.31*
**Fasted glycaemia (mg/dL)**	108.8 ± 2.55	175.17 ± 10.59***
**Fasted insulin serum (ng/mL)**	0.21 ± 0.13	3.32 ± 0.96*
**HOMA IR**	1.43 ± 0.87	33.36 ± 8.9***

Statistically significant: *p < 0.05; and ***p < 0.001. ND, normal diet; and HFD, high-fat diet.
