# Supplementary material for: Pancreatic alpha-cells from female mice undergo morphofunctional changes during compensatory adaptations of the endocrine pancreas to diet-induced obesity
Source: Sci Rep. 2015 Jun 25;5:11622. doi: 10.1038/srep11622 (PMC4650619; doi:10.1038/srep11622)
Supplement: Supplementary Information [file srep11622-s1.pdf]

SUPPLEMENTARY INFORMATION

Pancreatic alpha-cells from female mice undergo morphofunctional changes during compensatory adaptations of the endocrine pancreas to diet-induced obesity.

Beatriz Merino[1,2], Paloma Alonso-Magdalena[1,2], Mónica Lluesma[1,2], Patricia Ñeco[1,2], Alejandro Gonzalez[1,2,3], Laura Marroquí[1,2,4], Marta García-Arévalo[1,2], Angel Nadal[1,2], Ivan Quesada[1,2,*]

[1]*Instituto de Bioingeniería, Universidad Miguel Hernández, Elche, Spain.*
[2]*CIBER de Diabetes y Enfermedades Metabólicas Asociadas (CIBERDEM), Spain.*

[3]*Current address: Departamento de Biología, Universidad de Santiago de Chile, Santiago, Chile.*
[4]*Current address: Laboratory of Experimental Medicine, ULB Centre for Diabetes Research, Medical Faculty, Université Libre de Bruxelles, Brussels, Belgium.*

*to whom reprint requests should be addressed:
I. Quesada.
Instituto de Bioingeniería
Universidad Miguel Hernández
Avenida de la Universidad s/n
03202 Elche, Spain.
Phone: (+34) 96 522 2003
Email: ivanq@umh.es

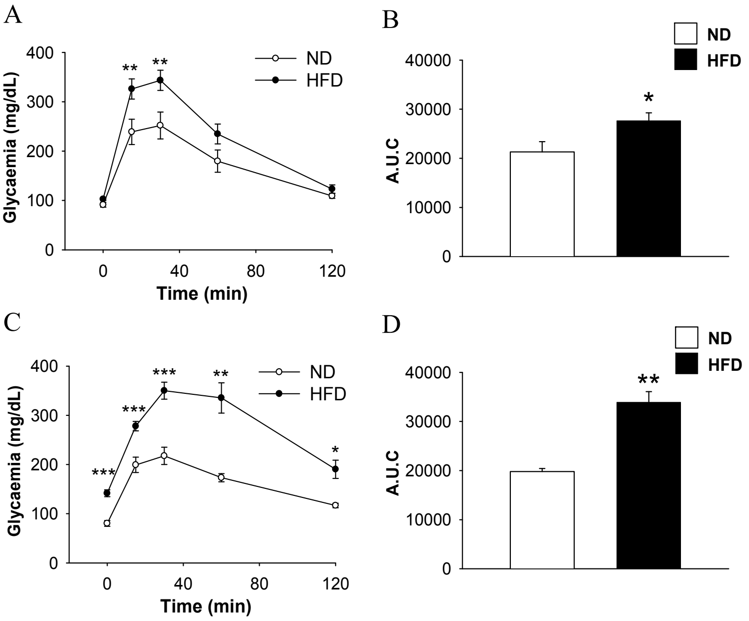

Supplementary Figure 1. **Glucose tolerance in ND and HFD animals after 12 and 24 weeks of diet.** A: Intraperitoneal glucose tolerance tests in animals after 12 weeks of treatment (n=6 mice per group). B: Area under curve (A.U.C.) of the glucose tolerance tests. C: Intraperitoneal glucose tolerance tests in animals after 24 weeks of treatment (n=5 mice for ND; n=10 mice for HFD). D: Area under curve of the glucose tolerance tests. Statistically significant: *p<0.05 **p<0.01; ***p<0.001. ND, normal diet; HFD, high-fat diet.

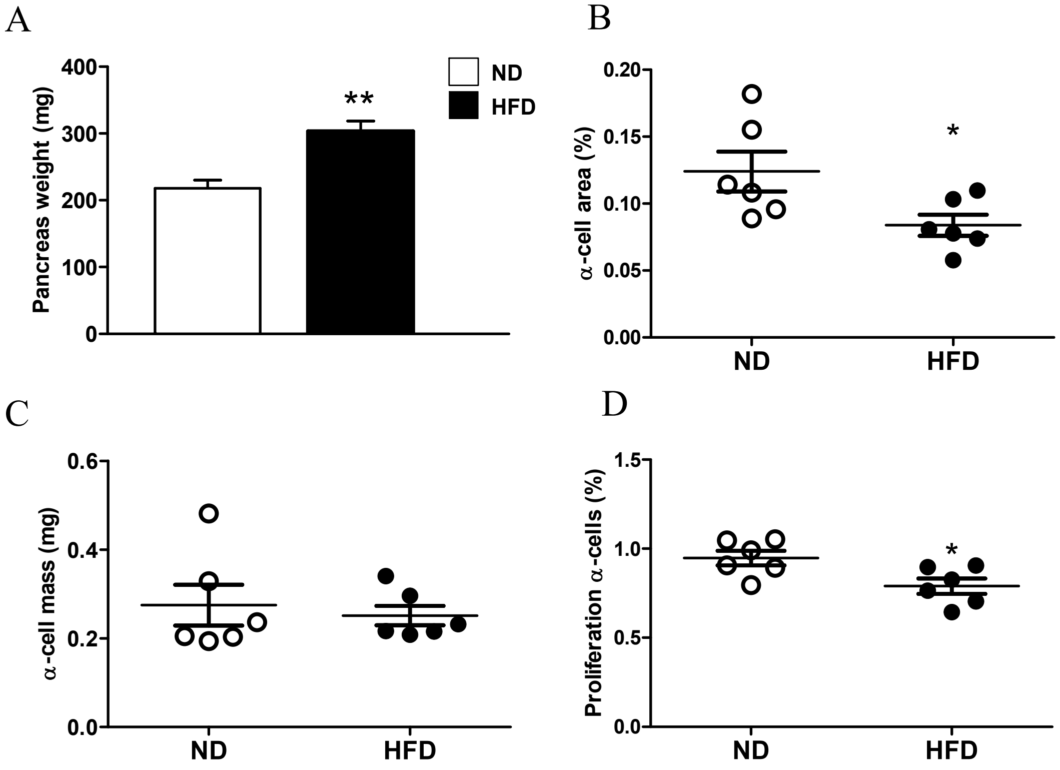

Supplementary Figure 2. **Alpha-cell morphological analysis in ND and HFD animals after 12 weeks of diet.** A: Pancreas weight (n=6 mice per group). B: Alpha cell area (%). C: Absolute alpha-cell mass. In B and C, six mice and two pancreas sections per animal were studied. D: Proliferation measured as percentage of glucagon-positive cells that incorporated BrdU (ND, n=2351 cells; HFD, n=2634 cells; 6 mice per condition). Statistically significant: *; p<0.05; **p<0.01. ND, normal diet; HFD, high-fat diet.

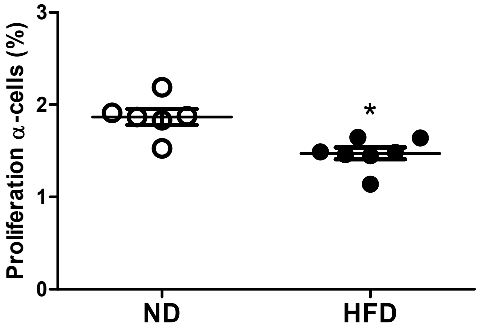

Supplementary Figure 3. **Alpha-cell proliferation measured by PCNA labelling in ND and HFD animals after 12 weeks of diet.** Proliferation measured as percentage of glucagon-positive cells that were labelled for PCNA (ND, n=3023 cells from 6 mice; HFD, n=3039 cells from 7 mice). Statistically significant: *; $p<0.05$. ND, normal diet; HFD, high-fat diet.
